# Sexual and reproductive health services for adolescents and young people with disabilities in Sub-Saharan Africa countries, 2013–2024: a scoping review

**DOI:** 10.1186/s13690-026-01987-z

**Published:** 2026-06-18

**Authors:** Luke Ojo, Samuel Oke, Monica Ansu-Mensah, Themba G. Ginindza, Desmond Kuupiel

**Affiliations:** 1 Discipline of Public Health, School of Medicine, College of Health Sciences, University of KwaZulu-Natal, Durban, 4001 South Africa; 2 Faculty of Health and Allied Sciences, School of Public Health, Catholic University of Ghana – Fiapre, Sunyani, Ghana; 3 Directorate of Grants, Research and Development, Sunyani Technical University, Sunyani, Ghana; 4 Cancer & Infectious Diseases Epidemiology Research Unit (CIDERU), College of Health Sciences, University of KwaZulu-Natal, Durban, 4001 South Africa; 5 Department of Nursing & Public Health, Faculty of Health Sciences, University of Fort Hare, East London, 5200 South Africa

**Keywords:** Adolescents, Young people, Disability, Sexual and reproductive health, Scoping review, Sub-Saharan Africa

## Abstract

**Background:**

Adolescents and young people with disabilities (AYPWDs) face significant inequities in sexual and reproductive health (SRH) service utilisation. This scoping review maps available SRC service, utilisation rates, barriers, facilitators and service quality across sub-Saharan Africa (SSA) to inform inclusive police development.

**Methods:**

This scoping review followed Arksey and O’Malley’s methodological framework, and was reported in accordance with Preferred Reporting Items for Systematic Reviews and Meta-Analysis modified for-Scoping Reviews guidelines. Five electronic databases (PubMed, Scopus, Web of Science, CINAHL, and PsycINFO) were searched using predefined keywords and Boolean operators. Titles, abstracts, and full texts were screened independently using predefined eligibility criteria. Data from included studies were extracted using a structured form and synthesised using descriptive and thematic analysis. Studies published between January 2013 and August 2024 were included in this review.

**Results:**

Sixteen studies were included. The studies took place in eight countries (greatest contributions from Ethiopia and Tanzania) with cross-sectional (56.25%), qualitative (25.00%), and mixed-methods (18.75%) study designs. The SRH services are grouped into prevention (family planning, STI/HIV prevention), care/treatment (maternal services, STI/HIV care), counselling/education (comprehensive sex education, counselling), and support services (GBV counselling, psychosocial support). Utilisation ranged from low to moderate, where assessed. Stigma/discrimination, negative provider attitudes, communication barriers (including the absence of sign language and adapted materials), inaccessibility, and weak confidentiality/privacy were the most frequently reported barriers. Key facilitators included education on reproductive health, peer and family support, positive provider attitudes, facility location, and sensitive communication with YPWD. Service quality was most commonly reported as poor, with limited privacy, long waiting times, and inadequate adaptation to be inclusive of people with disability.

**Conclusion:**

Maximising disability inclusion of the country's SRH policy and plans and Health system strengthening in consonance with the CRPD and the 2030 Sustainable Development Agenda is key to making equitable access a reality and to address structural and attitudinal barriers by building the capacity of providers, delivering services that are responsive to the needs of persons with disabilities, and making facilities and information progressively more accessible.

**Supplementary Information:**

The online version contains supplementary material available at https://doi.org/10.1186/s13690-026-01987-z.

**Table:** 

Text box 1. Contributions to the literature
• The review identifies there are persisting inequalities in the access to sexual and reproductive health (SRH) services among adolescents and young adults with disabilities in sub-Saharan Africa
• It offers the initial detailed synthesis of the available SRH services, utilization, as well as barriers and enhancers of access in the region
• The study points out the imperative for disability-inclusive SRH policies, training of health workers, and inclusive service environments
• Lessons of the review offer pragmatic recommendations to governments and other stakeholders in the strengthening of equity-focused SRH programs and universal health coverage

## Background

Sexual and reproductive health (SRH) is a fundamental component of overall health and human rights [1]. It encompasses physical, mental, and social well-being in matters relating to the reproductive system and includes access to information, education, and appropriate healthcare services [1]. Ensuring equitable access to SRH services is particularly important for vulnerable populations such as adolescents and young people with disabilities (AYPWDs), who often experience significant health disparities and social exclusion.

Disability is broadly defined as long-term physical, intellectual, sensory, or psychosocial impairments that, in interaction with environmental and societal barriers, may hinder full participation in society [2, 3]. Globally, an estimated 16% of the population lives with some form of disability, representing more than one billion worldwide [4]. Young people with disabilities are among the poorest and most marginalised of the world’s youth. Global estimates suggest that there are between 180 and 220 million youth with disabilities worldwide, and nearly 80 percent of them reside in low- and middle-income countries [4], where health systems face significant resource constraints.

Adolescence and young adulthood represent critical life stages characterised by rapid physical, emotional, and social development, during which access to accurate information and appropriate SRH services is essential. However, AYPWDs frequently encounter multiple barriers that limit their ability to obtain these services [5]. Historically, individuals with disabilities have often been incorrectly perceived as asexual or incapable of forming intimate relationships. Such misconceptions have contributed to their exclusion from SRH programmes and health education initiatives [6, 7]. Evidence increasingly demonstrates that adolescents and young people with disabilities have similar sexual and reproductive health needs as their non-disabled peers and require access to information and services that support healthy relationships, reproductive autonomy, and informed decision-making [8].

Despite these needs, access to SRH services for AYPWDs remains limited in many settings. Studies across different contexts indicate that young people with disabilities are at increased risk of adverse sexual and reproductive health outcomes, including unintended pregnancies, sexually transmitted infections (STIs), and gender-based violence [5, 9]. These disparities are often driven by a complex interplay of structural, socio-cultural, and health system barriers. For example, stigma and discriminatory attitudes towards persons with disabilities frequently discourage health-seeking behaviour and undermine their autonomy in making reproductive health decisions [10–12]. At the same time, health facilities may lack accessible infrastructure, disability-adapted communication tools, and trained providers capable of delivering inclusive and youth-friendly services.

The challenges faced by AYPWDs in accessing SRH services are particularly pronounced in sub-Saharan Africa (SSA), where health systems often operate under significant resource constraints. Socio-economic inequalities, cultural norms surrounding sexuality, and limited disability-inclusive policies further compound these challenges [13]. Evidence from individual country studies highlights the multifaceted nature of these barriers. In Senegal, for example, young people with disabilities reported low levels of knowledge and utilisation of contraception and gynaecological services, largely due to stigma and limited service accessibility [14]. Similarly, studies conducted in Ghana have documented inadequate access to contraceptive services among adolescents with disabilities despite relatively high levels of awareness about SRH issues [15]. In South Africa and other settings, negative attitudes among healthcare providers, lack of confidentiality, and limited disability-friendly communication methods have also been identified as key obstacles to equitable service delivery [9].

Beyond structural barriers within healthcare systems, broader social determinants further shape the SRH experiences of AYPWDs. These include poverty, limited educational opportunities, and social exclusion from mainstream youth programmes and services. Adolescents with disabilities are often excluded from sexuality education programmes and may lack supportive family or community environments that encourage open discussions about SRH issues [16, 17]. As a result, many young people with disabilities rely on informal or inaccurate sources of information, increasing their vulnerability to poor reproductive health outcomes. These challenges are compounded by persistent social stereotypes that portray persons with disabilities as sexually inactive or incapable of parenthood, thereby reinforcing their marginalisation from SRH policies and programmes [10–12].

Although an increasing number of studies have begun to explore SRH issues among persons with disabilities, the available evidence remains fragmented. Existing research in SSA varies widely in terms of geographical coverage, study design, and focus areas, often examining specific barriers or service experiences without providing a comprehensive synthesis of the broader evidence base. Consequently, it remains difficult for policymakers, practitioners, and researchers to obtain a consolidated understanding of the types of SRH services available to AYPWDs, the extent to which these services are utilised, and the factors influencing access and quality of care in the SSA region.

To address this gap, this scoping review aimed to map and synthesise the range of existing scientific evidence on sexual and reproductive health services for adolescents and young people with disabilities aged 10–24 years in sub-Saharan Africa. Specifically, the review examined the types of SRH services available, the extent of their utilisation, and the barriers, facilitators, and quality of SRH service provision experienced by this population. By systematically consolidating evidence across multiple settings, this review provides a clearer understanding of current knowledge gaps and offers an evidence base to inform disability-inclusive policies, health system strengthening, and future research priorities aimed at improving SRH outcomes for adolescents and young people with disabilities in SSA.

## Methods

This scoping review was conducted in accordance with the Arksey and O’Malley methodological framework [18], further refined by Levac et al. [19], and reported following the Preferred Reporting Items for Systematic Reviews and Meta-Analyses Extension for Scoping Reviews (PRISMA-ScR) guidelines. The scoping review methodology was selected because it enables a comprehensive mapping of existing evidence and facilitates systematic exploration of the breadth, nature, and extent of research on a given topic [20, 21]. In addition, scoping reviews are useful for identifying knowledge gaps, informing future systematic reviews, and highlighting priority areas for further primary research [22].

In line with Arksey and O’Malley’s framework [18], this review followed the five mandatory key steps:

- Identifying the research question
- Identifying relevant studies
- Selecting relevant studies
- Data charting
- Summarising and reporting the results

These steps are described in detail below.

### Identifying the research question

The main research question for this study was: What is the range of existing scientific evidence on SRHs for adolescents and young people with disabilities (ages 10–24) over the past decade in Sub-Saharan Africa? The sub questions included: (Table 1)

1. What types of SRH services are available for adolescents and young people with disabilities?
2. What is the extent of utilization of SRH services among adolescents and young people with disabilities?
3. What barriers and/ or facilitators influence their access to SRH services?
4. What is known about the quality of SRH services provided to this population?

**Table 1:** Population, Concept, and Context (PCC) framework defining the research question for a scoping review on sexual and reproductive health services for adolescents and young people with disabilities in Sub-Saharan Africa, 2013–2024

Population	This will include adolescents and young people aged 10–24 years with any form of disability (physical, intellectual, sensory, or multiple disabilities)
Concept	Sexual and reproductive health services, such as education, counselling, contraception, STI prevention and treatment, maternal health services, and other related services
Context	Sub-Saharan Africa (Countries with the World Health Africa Region)

*STI* Sexually Transmitted Infection

### Identifying relevant studies

A comprehensive search was conducted in multiple electronic databases, including EBSCOhost (CINAHL and PsycINFO), Web of Science, SCOPUS, Medline (PubMed), and Google Scholar (See Supplementary Table 1), to identify relevant studies published from January 2013 to August 2024. This time frame was chosen to capture the most recent decade of research, ensuring the inclusion of up-to-date evidence while reflecting current trends, policies, and advancements in sexual and reproductive health services for adolescents and young people with disabilities.

The search strategy was developed in collaboration with an expert librarian to ensure a comprehensive and systematic approach. Keywords such as 'adolescents,' 'young people,' 'disabilities,' 'sexual health,' 'reproductive health,' and 'services' were used, combined with Boolean operators (‘AND’ and ‘OR’) and Medical Subject Headings (MeSH) terms for optimal retrieval (Supplementary Table 1).

In addition to database searches, the World Health Organisation (WHO) website and reference lists of included studies were manually reviewed to identify additional relevant studies. No language restrictions were applied; however, only peer-reviewed studies that employed primary research methods (quantitative, qualitative, or mixed-methods) were included.

To ensure rigour, the search process was thoroughly documented, including search dates, keywords, and the number of eligible studies identified. A preliminary PubMed search was conducted to assess feasibility, and an expert librarian provided guidance throughout the process. All citations were managed using EndNote X20.

### Eligibility criteria and study selection

#### Eligibility criteria

The selection of studies for this scoping review was guided by predefined eligibility criteria. Studies were included if they focused on adolescents and young people with disabilities aged 10 to 24 years and addressed SRH services such as education, counselling, contraception, sexually transmitted infection (STI) prevention and treatment, maternal health services, and other related services.

Additionally, studies were eligible if they examined barriers to SRH access, including lack of knowledge, physical accessibility challenges, stigma, and shortages of trained providers. Studies identifying facilitators such as inclusive policies and tailored interventions were also included. Furthermore, the review considered studies reporting on SRH service outcomes, including health impacts, service quality, user satisfaction, and utilisation rates among adolescents and young people with disabilities.

While the initial search strategy considered a broad geographic range to ensure no relevant data were missed, the final inclusion criteria were strictly limited to studies conducted within SSA to maintain a focused regional synthesis. Studies examining service delivery models from public, private, and non-governmental organizations were also included.

To ensure methodological rigour, only peer-reviewed publications from January 2013 to August 2024 were considered, with a focus on English-language articles. The review included studies utilising quantitative, qualitative, and mixed-methods designs to provide a comprehensive analysis of the existing literature.

### Exclusion criteria

To maintain a clear focus on the research questions and ensure the rigour of the synthesis, the following exclusion criteria were applied:

- Secondary research, such as systematic reviews, scoping reviews, and meta-analyses, grey literature, conference abstracts and editorials were excluded to avoid the double-counting of primary data.
- Research conducted outside SSA and high-income countries was excluded to ensure the regional relevance of the findings to the specific socio-cultural and healthcare contexts of SSA.
- Studies published in languages other than English were excluded due to the resource constraints for professional translation.
- Studies published before January 2013 were excluded to ensure the review reflects the most recent decade of policy shift and healthcare advancement.

### Study selection

After removing duplicate articles from the EndNote library, a systematic two-phase screening process was conducted to select studies for inclusion in the scoping review.

1. In Phase one, three reviewers (Lo, SO, and MA-M) independently screened titles and abstracts against the inclusion criteria. Studies were categorised as ‘include or ‘exclude’ and any disagreements were resolved through collective discussion and consensus.
2. In Phase Two, the full texts of the remaining studies were independently reviewed by LO and MA-M. Discrepancies at this stage were adjusted by a third reviewer (DK) to ensure objectivity and rigour.

To access full-text articles that were not freely available online, the University of KwaZulu-Natal’s library services were used. Additionally, emails were sent to the corresponding authors of relevant publications to request full-text access for screening.

The screening process used a piloted Google Forms tool to ensure consistency and accuracy. To maintain transparency and documentation, the study selection process was illustrated using the Preferred Reporting Items for Systematic Reviews and Meta-Analyses (PRISMA-ScR) flow diagram.

### Data charting

A structured data extraction form was developed to ensure consistency and comprehensiveness. The principal investigator (LO) designed the form, which was reviewed by all co-authors to ensure it captured all relevant data. Before full data extraction, the form was pretested by LO, SO, and MA-M using ten randomly selected articles to assess its consistency.

The extracted data included:

- Study characteristics: Authors, year of publication, country, study design, and sample size.
- Population characteristics: Age range, type of disability, and other relevant demographics.
- Description of SRH services: Types of services, delivery settings, providers involved, and utilisation.
- Barriers and facilitators: Factors influencing access to SRH services.
- Other significant findings: Any additional relevant insights identified in the studies.

Data extraction was conducted independently by LO, SO, and MA-M, with any discrepancies resolved by a third author (DK).

### Summarising and reporting the results

The extracted data were collated and analysed using quantitative (descriptive) and narrative synthesis.

- Descriptive analysis summarised study characteristics and population demographics to provide an overview of the research scope.
- Thematic analysis identified key themes related to:
  - Types of sexual and reproductive health (SRH) services
  - Utilisation of SRH services
  - Barriers and facilitators influencing access
  - Quality of SRH services

Findings were presented in a narrative format, supported by tables and charts where relevant. The results were discussed in relation to policy implications, practice improvements, and future research directions. This study adhered to the PRISMA Extension for Scoping Reviews (PRISMA-ScR) guidelines to ensure rigour and transparency in reporting.

## Results

**Fig. 1:**
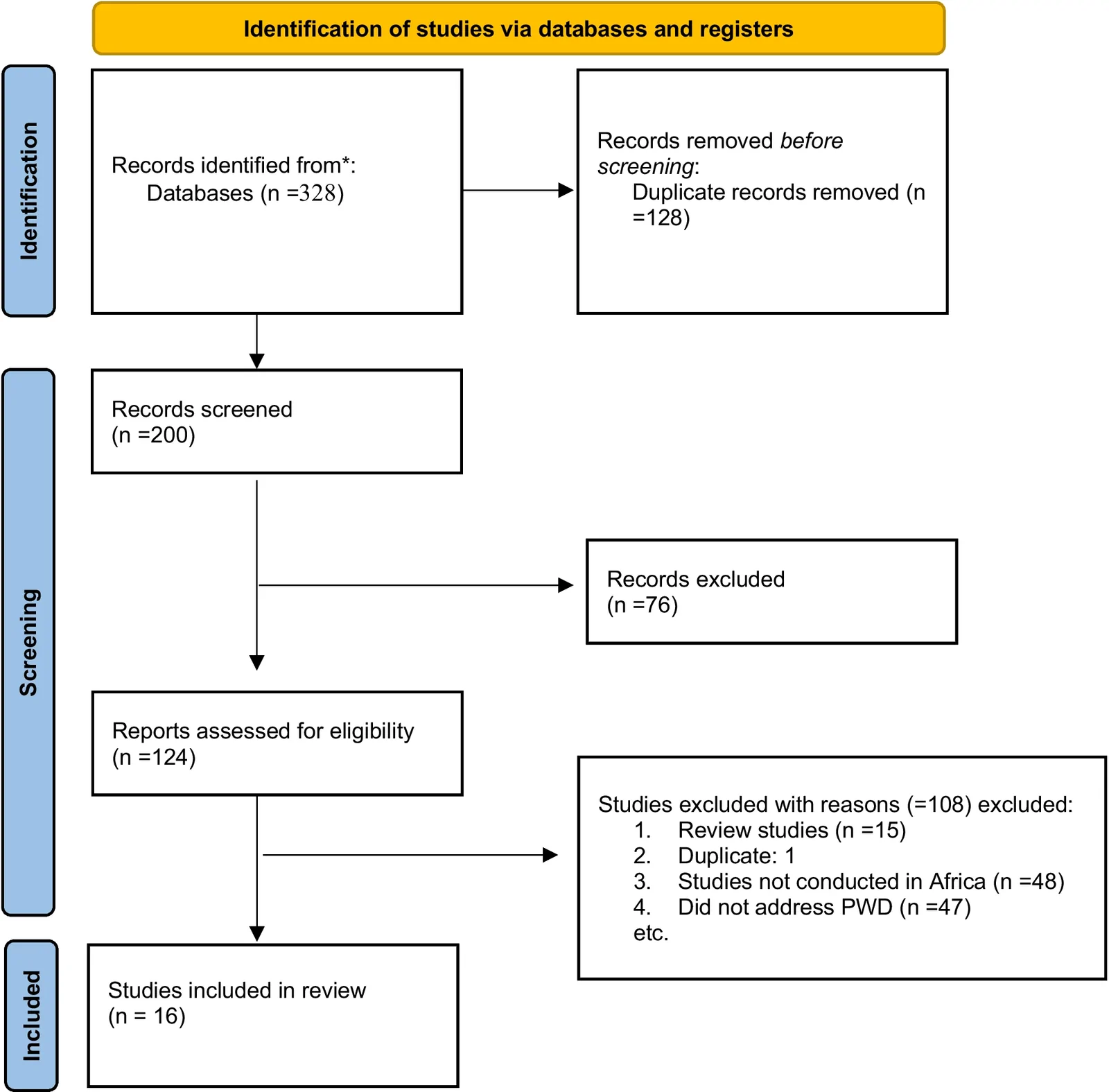
PRISMA-ScR Flow Diagram Showing Study Selection Process for a Scoping Review on Sexual and Reproductive Health Services for Adolescents and Young People with Disabilities in Sub-Saharan Africa, January 2013-August 2024

A total of 328 potentially eligible studies were initially identified from various databases, with no additional records found through other sources. After removing duplicates and screening against the eligibility criteria, 16 studies met the inclusion criteria and were included in the final synthesis (Fig. 1).

### Characteristics of the included studies

The final synthesis comprised 16 unique studies that specifically addressed or disaggregated data for adolescents and young people with disabilities (AYPWDs) in Sub-Saharan Africa [See Table 2]. Geographically, the evidence is heavily concentrated in East and West Africa, with Ethiopia (*n* = 4)[23–26], Ghana (*n* = 3) [27–29], and Tanzania (*n* = 3) [30–32] contributing the majority of the literature. Additional evidence was retrieved from Uganda (*n* = 2) [1, 8], Senegal (*n* = 1) [11], South Africa (*n* = 1) [34], and multi-country contexts (*n* = 1) [35]. Research interest has intensified recently, with 37.5% of papers published between 2022 and 2024 [8, 25, 26, 32–34], compared to 31.25% in 2019–2021 [27–29, 32, 35] and 31.25% in 2016–2018 [1, 3, 11, 23, 24]). Methodologically, cross-sectional quantitative designs predominated (56.25%)[1, 8, 23–25, 30, 32–34], The study populations were divided into adolescents with disabilities (37.5%), young people with disabilities (31.25%), and general youth samples (31.25%). Setting-wise, urban/tertiary facilities accounted for 37.5%, while strictly rural settings remained the most under-represented at 12.5% [See Table 2].

**Table 2:** Characteristics of 16 studies on sexual and reproductive health services for adolescent and young people with disabilities in Sub-Saharan Africa, 2013–2024

**Characteristics**	**n**	**(%)**
Study Country		
Ethiopia [23–26]	4	25.00%
Ghana [27–29]	3	18.75%
Tanzania [30–32]	3	18.75%
Nigeria [33]	1	6.25%
Uganda [1, 8]	2	12.5%
Senegal [11]	1	6.25%
South Africa [34]	1	6.25%
Multi-country (Zambia, Uganda, Ghana) [35]	1	6.25%
Year of Publication		
2016–2018 [1, 11, 23, 24, 30]	5	31.25%
2019–2021 [27–29, 32, 35]	5	31.25%
2022–2024 [8, 25, 26, 32–34]	6	37.50%
Study Design		
Cross-sectional quantitative [1, 8, 23–25, 30, 32–34]	9	56.25%
Qualitative [11, 27, 31, 35]	4	25.00%
Mixed-methods [26, 28, 29]	3	18.75%
Study Population		
Adolescents with disabilities [1, 8, 23–25, 33]	6	37.5%
Young people with disabilities [26, 29, 30, 32, 34]	5	31.25%
General youth (disaggregated for disability) [11, 27, 28, 31, 32]	5	31.25%
Study Settings		
Urban [23–25, 29, 33, 34]	6	37.5%
Rural [1, 11]	2	12.5%
Rural and Urban [8, 26, 30–32]	5	31.25%
Speciliased Settings (Special School/facilities) [27, 28, 35]	3	18.75%

### Types of SRH services accessed by adolescents and young people with disabilities reported across the included studies

The analysis of the 16 included studies revealed that sexual and reproductive health (SRH) services accessed by adolescents and young people with and without disabilities in SSA cluster into four broad themes](Supplementary Table 2]:

1. Prevention
  Family planning and contraceptives (condoms, pills, injectables, IUDs, implants, emergency contraception)
  HIV prevention services (condom use, HIV/AIDS education, voluntary counselling and testing [VCT])
  STI prevention (screening, risk-reduction education)
  Safe sex education (general awareness, menstrual health education)
  Violence prevention (gender-based violence [GBV] prevention, stigma reduction, psychosocial support)

**Fig. 2:**
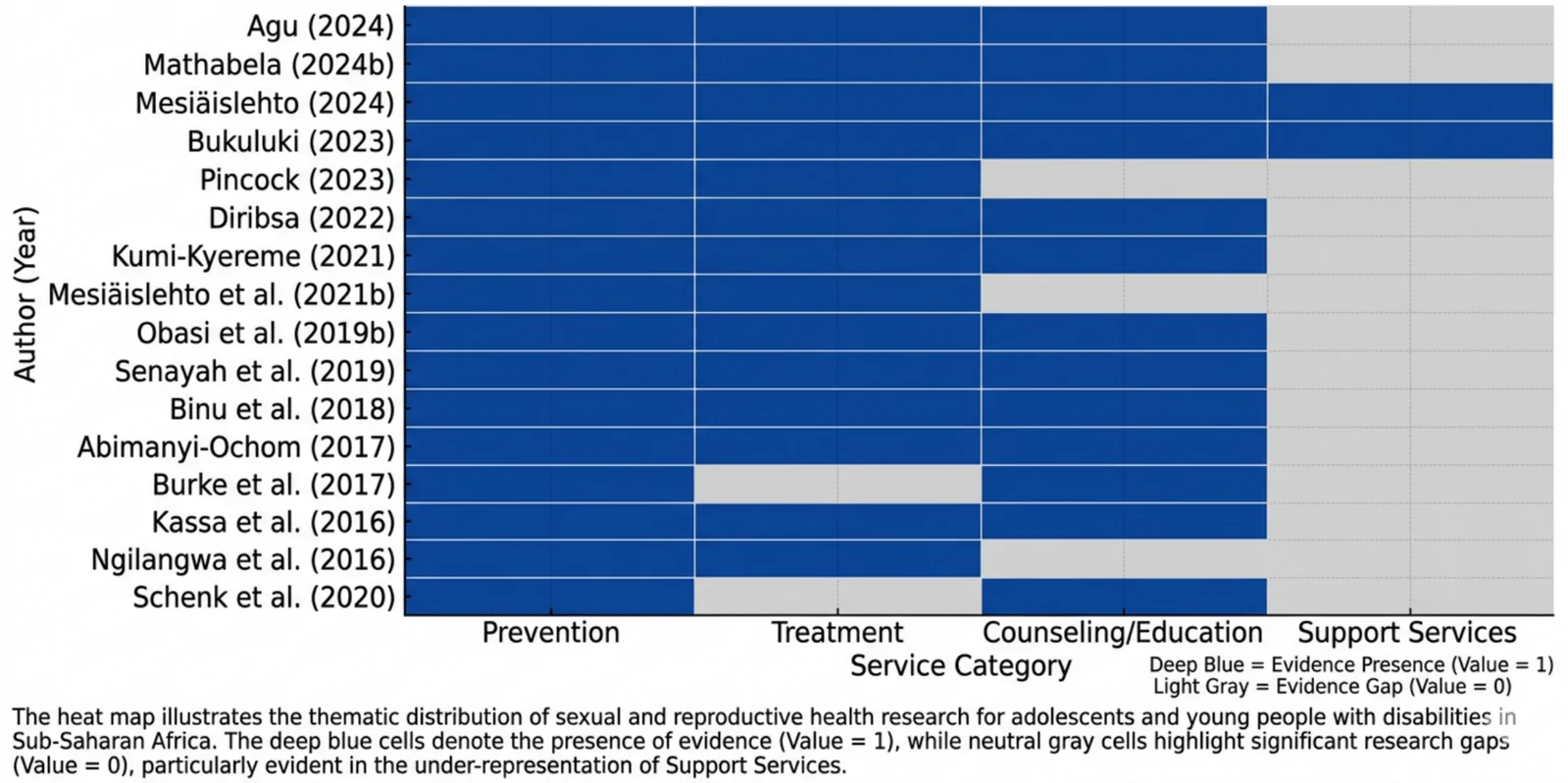
Evidence Map of Sexual and Reproductive Health (SRH) Services Available to Adolescents and Young People with Disabilities in Sub-Saharan Africa: Findings from a Scoping Review, 2013–2024

Prevention services were documented in all 16 studies [Fig. 2]. Key interventions included contraceptive provision (e.g., condoms, oral contraceptives, injectables, IUDs, and emergency pills) and family planning counselling [1, 8, 11, 23–35], and HIV/STI prevention through voluntary counselling and testing (VCT)and safe sex education [1, 11, 23]. A smaller number of studies reported preventive interventions for sexual and gender-based violence (SGBV) and stigma reduction [8, 31]

2. Treatment
  HIV testing, diagnosis, treatment, and care (including ART)
  STI testing, diagnosis, and treatment
  Maternal health services (antenatal care, gynecological consultations, maternal care)
  Abortion-related services (abortion counselling, post-abortion care, abortion service provision)

Treatment services were reported in 14 of the 16 studies. HIV testing, diagnosis, and treatment were the most frequently noted treatment-related services [8, 11, 23, 24, 30, 32–34]. STI treatment and management were also common [1, 24, 28, 29]. Maternal health services, including antenatal and gynecological care, were reported in four studies [8, 25, 31, 33], suggesting a narrower research focus. Abortion-related services (e.g., post-abortion care, abortion counselling) were reported in only two studies [26, 27], indicating a notable evidence gap.

3. Counselling and education
  General SRH education (sexuality education, comprehensive sexuality education [CSE], safe sex, menstrual health)
  Family planning and premarital counselling
  Counselling services tailored for adolescents and young people (including disability-inclusive adaptations and communication support for deaf adolescents)
  Risk behavior counselling (multiple partnerships, transactional sex, risky practices)
  Youth-friendly SRH counselling (tailored, age-appropriate services)

Counselling and education services were reported in 13 of the 16 studies, making this one of the most common service types. General SRH education covering safe sex practices, menstrual health, and comprehensive sexuality education (CSE) was widely emphasised [8, 11, 23, 24, 26, 33, 34]. Counselling services ranged from premarital and adolescent-focused counselling to disability-sensitive services, reflecting the importance of communication in SRH care delivery [1, 25, 27, 29, 31, 32].

4. Support services
  Gender-based violence (GBV) counselling and violence prevention
  Stigma reduction interventions in healthcare settings
  Psychosocial support services (for survivors of violence, marginalised groups, and persons with disabilities)

Support services were the least documented, reported in only 2 of the 16 studies. These focused on sexual and gender-based violence counselling, violence prevention, psychosocial support, and stigma reduction interventions [8, 31]. These findings emphasise the need for inclusive, youth-friendly, and disability-adapted interventions.

### Utilisation of SRH services among adolescents and young people with disabilities

Evidence on the utilisation of sexual and reproductive health (SRH) services among adolescents and young people in sub-Saharan Africa was reported in a limited number of studies, with uptake generally ranging from low to moderate. Reported SRH service utilisation ranged from 21.2% in Ethiopia to 42.0% in Northern Uganda [8, 23]. Frequently accessed services identified across these settings included family planning, voluntary HIV testing, and STI treatment [8, 23, 27, 30].

While quantitative uptake varied, studies consistently highlighted the specific types of SRH services accessed by this demographic. In Ghana, [27] documented the use of family planning and sexuality education among in-school AYPWDs, while in Tanzania, Ngilangwa et al. [30] similarly noted the use of contraceptives and HIV/AIDS education among marginalised youth. Despite the availability of these youth-friendly services, Agu et al. [33] emphasised that actual utilisation was significantly suppressed by pervasive stigma and discriminatory practices within clinical environments, a finding that underscores the persistent gap between service availability and inclusive access.

### Barriers to SRH services among adolescents and young people with disabilities

Barriers to accessing and utilising sexual and reproductive health (SRH) services among adolescents and young people with disabilities were highlighted across several studies [Table 3]. The findings demonstrate that, despite the availability of essential SRH services such as family planning, HIV testing and counselling, STI treatment, and sexuality education, multiple structural and social factors continue to limit equitable access.

**Table 3:** Barriers to Sexual and Reproductive Health (SRH) service access among adolescent and young people with disabilities in Sub-Saharan Africa: Evidence from a Scoping Review, 2013–2014

Author	Country	Key SRH Services Used	Noted Barriers
Kumi-Kyereme et.al. [27]	Ghana	Family planning, HIV counselling/testing, STI treatment, sexuality education/counselling	Limited disability-inclusive services
Ngilangwa et al. [30]	Tanzania	Contraceptives, HIV/AIDS education, family planning, STI treatment	Access constraints among marginalised youth
Agu et al. [33]	Nigeria	Contraceptives, HIV testing and counselling, STI treatment, abortion-related services	Stigma, discrimination, lack of youth-friendly-care

*STI* Sexually Transmitted Infection, *HIV* Human Immunodeficiency Virus

In Ghana, Kumi-Kyereme [27] reported that access to family planning and HIV counselling services was constrained by the absence of disability-inclusive service models, limiting effective participation of young people with disabilities in SRH programs. Similarly, Ngilangwa et al. [30] in Tanzania identified access constraints among marginalised youth, particularly those facing compounded barriers due to disability and social exclusion, despite the presence of contraceptive, HIV, and family planning services. In Nigeria, Agu et al. [33] highlighted that pervasive stigma, institutional discrimination, and a lack of youth-friendly clinical environments were major deterrents to service uptake. These findings are echoed across the broader synthesis, which identifies social stigma, discriminatory provider attitudes, and a systemic lack of disability-inclusive adaptations as the predominant barriers across sub-Saharan Africa [27, 30, 33]. This consistent evidence of exclusion suggests that merely increasing service availability is insufficient without addressing the underlying socio-cultural and structural biases that disenfranchise young people with disabilities.

### Facilitators to SRH service among adolescents and young people with disabilities

A limited number of studies identified key facilitators that enhanced access to and utilisation of sexual and reproductive health (SRH) services among adolescents and young people with disabilities (AYPWDs) in Sub-Saharan Africa [Table 4]. The findings suggest that access is positively influenced by a synergy of individual, interpersonal, and structural factors, including heightened awareness, supportive social networks, and proximity to health facilities. In Senegal, Burke et al. [11] reported that peer-led support and the involvement of community health workers, combined with the geographical proximity of health facilities, significantly facilitated access to contraception, gynecological consultations, and antenatal services. In Uganda, Abimanyi-Ochom et al. [1] highlighted the critical role of self-efficacy in motivating AYPWDs to seek contraception, STI testing, and counseling services.

**Table 4:** Facilitators of Sexual and Reproductive Health (SRH) service access among adolescents and young people with disabilities in Sub-Saharan Africa: Evidence from a Scoping Review, 2013–2024

Author(s)	Country	Key SRH services used	Noted facilitator
Burke et al., [11]	Senegal	Contraception, gynecological consultations, antenatal services	Support from peers and community health workers. Closer health facilities are more accessible
Abimanyi-Ochom et al. [1]	Uganda	Contraception, STI testing, and counseling	Promoting self-efficacy
Kassa [24]	Ethiopia	Family planning, STI testing, HIV counseling and testing, gynecological consultation	Radio and TV: Main sources of SRH information
Senayah [28]	Ghana	General health services, including SRH services	Friendly attitudes of health professionals. Support from family members

*SRH* Sexual and Reproductive Health, *AYPWD* Adolescents and Young People with Disabilities, *SSA* Sub-Saharan Africa

In Ethiopia, Kassa et al. [24] found that consistent exposure to SRH information through mass media, such as radio and television, enhanced awareness and encouraged the utilisation of family planning and STI testing services. Similarly, in Ghana, Senayah et al. [28] reported that the friendly attitudes of healthcare professionals and supportive family relationships were pivotal in encouraging adolescents with disabilities to access general and specialised SRH services.

### Quality of service among adolescents and young people with disabilities

The quality of sexual and reproductive health (SRH) services available to adolescents and young people with disabilities (AYPWDs) was consistently described as poor across the majority of the reviewed settings [Table 5]. Evidence indicates systemic deficiencies in privacy, confidentiality, physical accessibility, and provider attitudes. In Senegal, Burke et al. [11] identified critical gaps in confidentiality and noted unsupportive healthcare worker behaviours during the provision of contraception, antenatal, and gynaecological services. Similarly, in Ethiopia, Kassa et al. [24] reported that physical inaccessibility of facilities and a lack of private consultation spaces significantly compromised the quality of family planning and HIV testing services.

**Table 5:** Quality of Sexual and Reproductive Health (SRH) services for adolescents and young people with disabilities in Sub-Saharan Africa: Evidence from a Scoping Review, 2013–2024

**Author(s)**	Setting/Country	**Key SRH services used**	**Quality of Service and Noted Issues**
Obasi et al. [29]	Ghana	Information on SRH, contraceptives (mainly condoms), HIV testing	Poor: Lack of knowledge about contraceptives, weak provider engagement
Burke et al., [11]	Dakar, Thies, and Kaolack, Senegal	Contraception, gynecological consultations, antenatal services	Poor: Lack of confidentiality, negative health worker attitudes
Kassa [24]	Addis Ababa, Ethiopia	Family planning, STI testing, HIV counseling/testing, gynecological consultation	Poor: Physical inaccessibility and lack of confidentiality
Schenk et al., [35]	South Africa	HIV services, counseling, ART	Poor, due to compounded stigma, misinformation, and attitudinal barriers among healthcare providers
Senayah [28]	Ghana	Family planning, HIV testing, counseling	Poor: Due to communication barriers and lack of privacy
Agu et al.,. [33]	Nigeria	Contraceptives, STI treatment, HIV counseling	Generally harsh and unfriendly, with variations depending on the young person’s social identity

*SRH* Sexual and Reproductive Health, *AYPWD* Adolescents and Young People with Disabilities, *SSA* Sub-Saharan Africa

Comparable patterns were observed in Ghana and Uganda. Obasi et al. [29] noted that AYPWDs in Ghana experienced inadequate provider engagement and received insufficient information regarding contraceptives and HIV testing. In Uganda, Abimanyi-Ochom et al. [1] reported a pervasive lack of confidentiality and negative staff attitudes as primary factors discouraging the utilisation of STI and contraceptive services. Furthermore, Senayah et al. [28] highlighted that communication barriers between providers and hearing-impaired adolescents in Ghana, coupled with a lack of privacy, severely undermined the quality of care.

In a multi-country context involving Zambia, Uganda, and Ghana, Schenk et al. [35] described HIV-related services as being of poor quality, characterised by compounded stigma and misinformation among healthcare providers. These findings are echoed by Agu et al. [33] in Nigeria, who reported that SRH service environments were often exclusionary, with provider support varying based on the young person’s perceived vulnerability. Collectively, the evidence underscores that negative provider attitudes, lack of confidentiality, and communication barriers remain the dominant factors contributing to poor SRH service quality.

## Discussions

To provide a structured synthesis of the evidence, the following discussion is organised into four key thematic areas identified across the included studies. First, the study examined the utilisation rates of SRH services among adolescents and young people with disabilities (AYPWDs). This is followed by an analysis of the multi-level barriers and enabling of services and systemic gaps currently affecting the inclusivity and responsiveness of SRH provision in Sub-Saharan Africa.

### Utilisation of SRH services among adolescent and young people with disabilities

The findings of this synthesis reveal that the utilisation of sexual and reproductive health (SRH) services among adolescents and young people with disabilities (AYPWDs) across Sub-Saharan Africa remains critically suboptimal. Quantitative evidence suggests a narrow uptake range, from a low of 21.2% in Ethiopia to approximately 42.0% in Uganda [8, 23]. This utilisation gap is significant when compared to general youth populations in the region, suggesting that disability remains a compounding barrier to healthcare engagement. While clinical services such as HIV testing, STI management, and contraceptive provision were the most frequently accessed [23, 28, 30], the overall "low-to-moderate" uptake across all 16 studies indicates that service availability does not equate to service accessibility.

The patterns of service use identified in Ghana, Tanzania, and Nigeria [27, 30, 33], underscore a global health paradox: while AYPWDs recognise the presence of "youth-friendly" services, actual engagement is suppressed by pervasive environmental and attitudinal barriers. For instance, the emphasis on family planning and HIV/STI services in the literature reflects the heavy donor-driven prioritisation of these areas in Sub-Saharan Africa, often at the expense of more holistic support services such as psychosocial counselling or violence prevention [8, 31].

Furthermore, the data suggest that stigma and institutional discrimination serve as primary deterrents, particularly in clinical settings where providers may lack disability-sensitive training. The reliance on facility-only or urban-centric service models also risks further marginalising the most vulnerable AYPWDs, those in rural areas or those out of school, who accounted for the lowest representation in our reviewed literature. Consequently, addressing the utilisation gap requires a paradigm shift from traditional "one-size-fits-all" adolescent health models toward integrated, disability-inclusive frameworks that address the intersectional barriers of age, disability, and geography.

### Barriers to SRH services among adolescents and young people with disabilities

The synthesis of the 16 included studies reveals a profound "implementation gap" across Sub-Saharan Africa, where the theoretical availability of essential services, such as family planning, HIV testing, and sexuality education, fails to translate into equitable access for adolescents and young people with disabilities (AYPWDs) (Table 4). This disconnect is primarily driven by a complex interplay of multi-level barriers that effectively marginalise AYPWDs from mainstream healthcare[(27,30,35)], In Ghana, Kumi-Kyereme [27] reported that the absence of disability-inclusive service models acts as a systemic deterrent, a finding that corroborates previous evidence suggesting that policy advancements often fail to reach the ground level if health system constraints and social attitudes are not addressed as a priority.

A critical theme emerging from this analysis is the "triple discrimination" faced by disabled young women, where age, gender, and disability intersect to create profound vulnerabilities. In Tanzania, Ngilangwa et al. [30] identified that marginalised youth face compounded barriers due to this intersectionality, leading to social exclusion that distances them from contraceptive and family planning services. These findings align with the socio-ecological model, which posits that barriers at the individual, interpersonal, and institutional levels interact to determine health outcomes. This is particularly evident in Nigeria, where Agu et al. [33], noted that pervasive stigma and discriminatory provider attitudes serve as major deterrents, reflecting deeply entrenched social biases that transcend geographic boundaries.

Furthermore, institutional barriers, including the physical inaccessibility of facilities, lack of sign language interpretation, and the absence of private, confidential consultation spaces, remain dominant inhibitors [24]. Such findings underscore a widespread regional failure to meet the WHO’s Availability, Accessibility, Acceptability, and Quality (AAAQ) framework. In fact, global evidence suggests that institutional barriers, specifically negative provider attitudes, are often more restrictive than physical ones, as they create an environment of "perceived exclusion" that discourages future health-seeking behaviour.

Collectively, the evidence demonstrates that stigma, discrimination, and a lack of inclusive adaptation are not isolated incidents but represent a systemic exclusion of AYPWDs from the regional health agenda [27, 30, 33], As noted in the Africa CDC Reproductive Health Strategy 2022–2026, achieving Universal Health Coverage (UHC) will remain unattainable without a paradigm shift that incorporates the voices of young people with disabilities into the very design of health policy and infrastructure. Without deliberate efforts to dismantle these attitudinal and informational barriers, AYPWDs will continue to navigate "intricate pathways" in the grip of many intersecting factors, perpetually lagging behind their non-disabled peers in the achievement of Sustainable Development Goals 3 and 10.

### Facilitators to SRH service among adolescents and young people with disabilities

While barriers are pervasive, this synthesis identified a synergistic set of individuals, interpersonal, and structural facilitators that enhance SRH service uptake among AYPWDs (Table 5). A critical finding across the 16 included studies is that access is significantly bolstered by supportive social networks and community-based interventions. In Senegal, Burke et al. [11] demonstrated that the involvement of peer educators and community health workers, combined with the proximity of facilities, created an enabling environment for utilisation of gynaecological and antenatal services. This aligns with the Health Belief Model, which suggests that "cues to action", such as peer support and proximity, are essential for translating health intent into actual behaviour. These findings are corroborated by broader evidence from Sub-Saharan Africa suggesting that community-embedded health workers act as vital "navigators" who bridge the trust gap between marginalised youth and formal clinical institutions [31, 35].

At the individual level, psychological factors such as self-efficacy and health literacy emerged as potent drivers of utilisation. In Uganda, Abimanyi-Ochom et al. [1] highlighted that adolescents with higher self-efficacy were significantly more motivated to seek STI testing and counselling, a finding corroborated by global evidence suggesting that empowerment-based interventions are more effective than purely clinical ones. Furthermore, the role of mass media in bridging the information gap was underscored by Kassa et al. [24] in Ethiopia, where consistent exposure to SRH messaging via radio and television was positively associated with family planning uptake. This suggests that disability-sensitive communication strategies (e.g., closed captioning or sign-language-integrated media) are indispensable for reaching marginalised youth. The effectiveness of such media-led interventions is further supported by Diribsa et al. [25], who noted that digital and visual health communication can bypass traditional literacy barriers often faced by adolescents with certain disabilities.

Institutionally, healthcare providers' attitudes remain the most influential structural facilitator. In Ghana, Senayah et al. [28] reported that "friendly" and "non-judgmental" provider interactions were pivotal in encouraging hearing-impaired adolescents to utilise general SRH services. This corroborates findings from the Triggerise "Digital Frontier" initiative, which emphasizes that adolescent-centered, stigma-free environments are the cornerstone of successful SRH programs. Additionally, Mathabela et al. [34] found that specialised training for providers on disability etiquette and rights-based care significantly reduced "perceived exclusion," making clinical spaces more inviting for young people with diverse needs. Collectively, the evidence indicates that while clinical infrastructure is necessary, interpersonal support and positive provider engagement are the primary catalysts for inclusive SRH care. Strengthening these enabling factors through targeted provider training and community-led advocacy could substantially reduce the persistent inequities documented in Fig. 2.

### Quality of service among adolescents and young people with disabilities

The quality of sexual and reproductive health (SRH) services for adolescents and young people with disabilities (AYPWDs) in Sub-Saharan Africa remains critically suboptimal across multiple dimensions [Table 5]. Evidence from the 16 included studies reveals that systemic deficiencies in privacy, confidentiality, and physical accessibility consistently compromise the quality of care. In Senegal, Burke et al. [11] found that poor confidentiality and unsupportive provider behaviours significantly hampered the quality of contraceptive and gynaecological consultations. These institutional barriers are mirrored in Ethiopia, where Kassa et al. [24] identified that physical inaccessibility of clinics and a lack of private consultation spaces served as primary indicators of poor service quality, particularly for sensitive services like HIV testing.

The "interpersonal quality" of care, specifically provider–client engagement, is another major area of concern. In Ghana, Obasi et al. [29] observed that adolescents with disabilities faced inadequate engagement from healthcare workers and received insufficient information regarding SRH options, creating a significant "information gap". Similarly, in Uganda, Abimanyi-Ochom et al. [1] noted that the overall quality of care was marred by a pervasive lack of confidentiality and judgmental staff attitudes, which acted as a strong deterrent for young people seeking STI and contraceptive services. Furthermore, Senayah et al. [28] highlighted that communication barriers, particularly for hearing-impaired youth in Ghana, often led to a total breakdown in service quality due to the absence of sign language interpretation and the resulting loss of patient privacy [36].

The persistent "quality gap" is often exacerbated by deep-seated stigma and misinformation within the clinical workforce. In a multi-country context involving Zambia, Uganda, and Ghana, Schenk et al. [35] described HIV-related services as being of poor quality, characterised by compounded stigma and provider-led misinformation regarding the sexual rights of people with disabilities. These findings are further corroborated by Agu et al. [33] in Nigeria, who reported that SRH service environments were often exclusionary, with the level of support provided by clinicians varying based on a young person’s perceived vulnerability. Collectively, this evidence suggests that a lack of provider sensitivity and infrastructural adaptation are the dominant factors contributing to poor-quality SRH services across the region. Addressing these gaps requires moving toward disability-sensitive quality assurance frameworks and integrating universal design principles to ensure that healthcare environments are truly inclusive and responsive to the rights of all AYPWDs.

### Implications for policy and practice

The results of this review have implications for improving SRH outcomes among adolescents and young people with disabilities. First, there is a critical need to mainstream disability inclusion within national SRH policies and strategies to ensure equitable access and participation. This can be achieved by incorporating disability-sensitive targets and indicators consistent with the CRPD and the 2030 Agenda for Sustainable Development.

Second, health system strengthening is necessary to address structural and attitudinal barriers that hinder SRH service delivery. Governments and partners should prioritise training healthcare providers on disability inclusion, communication (including sign language), and rights-based, respectful care. Evidence indicates that provider sensitisation improves accessibility, client satisfaction, and service uptake among persons with disabilities [36]. Additionally, health facilities must be made physically accessible, private, and youth-friendly, with adapted information and communication materials.

Third, community and peer-based approaches should be scaled up to promote awareness, self-efficacy, and empowerment among adolescents with disabilities. Such interventions have been shown to counter stigma and encourage positive health-seeking behaviour [17]. Finally, disability-disaggregated data should be integrated into national health information systems to guide planning, resource allocation, and evaluation of inclusive SRH programs. Strengthening these systems is essential for measuring progress toward SDG 3 and SDG 10 and for ensuring that no one is left behind.

### Strengths and limitations

This scoping review offers a comprehensive synthesis of SRH services for adolescents and young people with disabilities in sub-Saharan Africa. Its methodological rigour was guided by the Arksey and O’Malley [18] framework, refined by Levac et al. [19], and reported using PRISMA-ScR enabled systematic identification and mapping of diverse evidence. The inclusion of studies addressing service utilisation, barriers, facilitators, and quality provides a holistic understanding of SRH service delivery gaps and opportunities.

However, limitations exist. The evidence base remains relatively small, with most studies concentrated in a few countries, limiting regional generalizability. The review captures evidence up to August 2024 and may not reflect studies published thereafter. Variations in study design, populations, and outcome measures constrained direct comparison or meta-analysis. Additionally, the reliance on self-reported data in most studies may have introduced recall or desirability bias. The review’s focus on English-language studies may also have excluded relevant francophone or lusophone evidence. Despite these limitations, the review provides a valuable foundation for future research and policy development on inclusive SRH service delivery.

## Conclusion

This scoping review revealed that although a range of SRH services, such as family planning, HIV and STI testing and treatment, and counselling, are available, utilisation among adolescents and young people with disabilities remains low to moderate. Persistent barriers, including stigma, discrimination, poor provider attitudes, communication challenges, and physical inaccessibility, continue to impede equitable access. Conversely, facilitators such as health education, family and peer support, positive attitudes of healthcare providers, and proximity of facilities were shown to improve service uptake. There is a need to institutionalise disability-inclusive interventions, strengthen health system responsiveness, and promote rights-based provider training.

## Supplementary Information


Supplementary Material 1.


## Data Availability

No datasets were generated or analysed during the current study.

## Abbreviations

AYPWDs: Adolescent and Young People with Disabilities
SRH: Sexual and Reproductive Health
SSA: Sub-Saharan Africa
CRPD: Convention on the Rights of Persons with Disabilities
UHC: Universal Health Coverage
SDG: Sustainable Development Goal
PRISMA-ScR: Preferred Reporting Items for Systematic Review and Meta-Analyses extension for Scoping Reviews
