# Supplementary material for: Sexual and reproductive health services for adolescents and young people with disabilities in Sub-Saharan Africa countries, 2013–2024: a scoping review
Source: Arch Public Health. 2026 Jun 18;84:194. doi: 10.1186/s13690-026-01987-z (PMC13523526; doi:10.1186/s13690-026-01987-z)
Supplement: Supplementary file 1 [file 13690_2026_1987_MOESM1_ESM.docx]

**Supplementary material**

**Supplementary Table 1:** Search Strategy Used Across Five Electronic Databases for a Scoping Review on Sexual and Reproductive Health Services for Adolescents and Young People with Disabilities in Sub-Saharan Africa, January 2013 – August 2024

| Date | Database | Keyword | Search Result | Potentially eligible studies following titles screening |
| --- | --- | --- | --- | --- |
| 8/08/24 | Medline (PubMed), | Search #1: (adolescent with disability) OR (young people with disability)  Search #2: (Sexual health service) OR (reproductive health service)  Search #3: (((adolescent with disability) OR (young people with disability)) AND (Sexual health service)) OR (reproductive health service)  Search #4: (adolescent with disability OR young people with disability) AND (Sexual health service OR reproductive health service) | 13,876 results  90,645 results  66,691 results  403 | 53 |
| 14/08/2024 | Google Scholar | Search 1; Adolescents OR Young People AND Disabilities AND Sexual and Reproductive Health AND Accessibility AND Barriers  Search 2: Adolescents OR Young People AND Disabilities AND Sexual health OR Reproductive Health AND Accessibility AND Barriers. Filtered 2013-2024  Searches 3: Adolescents OR Young People AND Disabilities AND Sexual health OR Reproductive Health AND Accessibility AND Barriers AND Africa. Filtered 2013-2024 | 39,600 results  17,300 results  About 17,900 | 27 |
| 14/08/2024 | EBSCOhost (CINAHL and PsychINfo) | Search #1: adolescent with disability OR young people with disability  Search 2: (adolescent with disability OR young people with disability) AND (Sexual health service OR reproductive health service)  Search 3: (adolescent with disability OR young people with disability) AND (Sexual health service OR reproductive health service). Filter 2013-2024 | 13,234  21,023  12,699 | 173 |
| 15/08/2024 | Web of Science | (((((((ALL=(Adolescents)) OR ALL=(Young People )) AND ALL=(Disabilities )) OR ALL=(Disable)) AND ALL=(Sexual health )) OR ALL=(Reproductive Health )) AND ALL=(Accessibility)) AND ALL=(Barriers )  (((((((ALL=(Adolescents)) OR ALL=(Young People )) AND ALL=(Disabilities )) OR ALL=(Disable)) AND ALL=(Sexual health )) OR ALL=(Reproductive Health )) AND ALL=(Accessibility)) AND ALL=(Barriers ) Filter 2013-2024 | 313  280 | 36 |
|  | SCOPUS | ALL ( "Adolescents" ) OR ( "Young People" ) AND ( "Disabilities" ) OR ( "Disable" ) AND ( "Sexual health" ) OR ( "Reproductive Health" ) AND ( "Accessibility" ) AND ( "Barriers" )  ALL ( "Adolescents" ) OR ( "Young People" ) AND ( "Disabilities" ) OR ( "Disable" ) AND ( "Sexual health" ) OR ( "Reproductive Health" ) AND ( "Accessibility" ) AND ( "Barriers" ) AND PUBYEAR > 2012 AND PUBYEAR < 2025. Filtered 2013-2024 | 656  574 | 39 |
| **TOTAL POTENTIAL ELIGIBLE STUDY** | | | | **328** |

**Supplementary Table 2:**  Themes and Sub-themes of Sexual and Reproductive Health (SRH) Services Identified from a Scoping Review on Adolescents and Young People with Disabilities in Sub-Saharan Africa, 2013–2024.

| **Theme** | **Author and year** | **Key SRH services captured** | **Sub-category** |
| --- | --- | --- | --- |
| **Prevention** | Obasi et al. (2019) | Contraceptives (condoms, emergency pills), family planning counseling, SRH information | Family planning/contraceptives |
|  | Burke et al. (2017) | Contraceptive services (condoms, pills, IUDs, implants) | Family planning/contraceptives |
|  | Bukuluki (2023) | Modern contraceptives, HIV/STI prevention information, and risky behaviour services | Family planning/contraceptives; HIV/STI prevention |
|  | Kassa (2016) | Family planning methods, HIV prevention, safe sex education | Family planning/contraceptives; HIV/STI prevention |
|  | Senayah (2019) | HIV/STI testing and prevention, SRH education | HIV/STI prevention |
|  | Schenk et al. (2020) | HIV prevention education, stigma reduction, GBV prevention counseling | HIV/STI prevention; Violence prevention |
|  | Binu et al. (2018) | Family planning, HIV VCT, STI prevention, SRH counseling | Family planning/contraceptives; HIV/STI prevention |
|  | Agu et al. (2024) | Contraceptives, HIV prevention/testing, STI prevention, abortion-related | Family planning/contraceptives; HIV/STI prevention |
|  | Mesiäislehto (2024) | HIV testing, STI prevention, adolescent counseling | HIV/STI prevention |
|  | Diribsa et al. (2022) | Family planning, HIV VCT, STI prevention, post-abortion care | Family planning/contraceptives; HIV/STI prevention |
|  | Mathabela et al. (2024) | Contraceptives, HIV counseling/testing, STI prevention | Family planning/contraceptives; HIV/STI prevention |
|  | Mesiäislehto et al. (2021) | Family planning, HIV prevention/treatment, STI prevention | Family planning/contraceptives; HIV/STI prevention |
|  | Abimanyi-Ochom et al. (2017) | HIV prevention education, HIV counseling/testing, STI prevention | HIV/STI prevention |
|  | Ngilangwa et al. (2016) | Contraceptives, HIV/AIDS education, STI prevention, family planning | Family planning/contraceptives; HIV/STI prevention |
|  | Pincock et al. (2023) | Comprehensive sexuality education (CSE), HIV/STI prevention, violence prevention | SRH education; HIV/STI prevention; Violence prevention |
|  | Kumi-Kyereme (2021) | Family planning, HIV counseling/testing, STI prevention, sexuality education | Family planning/contraceptives; HIV/STI prevention |
| **Treatment** |  |  |  |
|  | Burke et al. (2017) | Gynecological consultations | Maternal health |
|  | Kassa (2016) | HIV prevention services, STI treatment | HIV/STI treatment |
|  | Senayah (2019) | HIV/STI testing and treatment | HIV/STI treatment |
|  | Schenk et al. (2020) | HIV testing, diagnosis, treatment, care | HIV/STI treatment |
|  | Binu et al. (2018) | STI treatment | HIV/STI treatment |
|  | Agu et al. (2024) | HIV testing, STI treatment, abortion services | HIV/STI treatment; Abortion-related |
|  | Mesiäislehto (2024) | Maternal care, STI treatment, HIV testing | Maternal health; HIV/STI treatment |
|  | Diribsa et al. (2022) | STI treatment, post-abortion care | HIV/STI treatment; Abortion-related |
|  | Mathabela et al. (2024) | STI treatment, HIV counseling/testing | HIV/STI treatment |
|  | Mesiäislehto et al. (2021) | Maternal care, HIV treatment, STI treatment | Maternal health; HIV/STI treatment |
|  | Abimanyi-Ochom et al. (2017) | HIV testing/treatment, STI treatment | HIV/STI treatment |
|  | Ngilangwa et al. (2016) | STI treatment, family planning | HIV/STI treatment |
|  | Pincock et al. (2023) | STI treatment | HIV/STI treatment |
|  | Kumi-Kyereme (2021) | STI treatment, HIV counseling/testing | HIV/STI treatment |
| **Counseling and Education** |  |  |  |
|  | Obasi et al. (2019) | SRH information, family planning counseling | SRH education |
|  | Burke et al. (2017) | SRH education and awareness | SRH education |
|  | Bukuluki (2023) | HIV/STI information, risky behavior counseling | SRH education/counseling |
|  | Kassa (2016) | General SRH education (safe sex, menstrual health) | SRH education |
|  | Senayah (2019) | General SRH counseling, communication services | SRH counseling |
|  | Schenk et al. (2020) | SRH counseling tailored for PWD | SRH counseling |
|  | Binu et al. (2018) | SRH information and counseling | SRH counseling |
|  | Agu et al. (2024) | Youth-friendly SRH counseling | SRH counseling |
|  | Mesiäislehto (2024) | Adolescent counseling | SRH counseling |
|  | Diribsa et al. (2022) | SRH education and counseling | SRH education/counseling |
|  | Pincock et al. (2023) | Comprehensive sexuality education | SRH education |
|  | Kumi-Kyereme (2021) | Sexuality education and counseling | SRH education/counseling |
| **Support Services** | Schenk et al. (2020) | GBV counseling, stigma reduction | Violence/GBV support; Stigma reduction |
|  | Pincock et al. (2023) | Violence prevention and psychosocial support | Violence/GBV support |

***Note: SRH = Sexual and Reproductive Health; STI = Sexually Transmitted Infection; HIV = Human Immunodeficiency Virus; GBV = Gender-Based Violence***

**Supplementary Table 3: List of Included Studies**

| No. | Author (Year) | Setting/Country | Study Design | Disability Focus | Key Findings & SRH Services |
| --- | --- | --- | --- | --- | --- |
| 1 | Mathabela et al. (2024) | South Africa | Qualitative | Physical | adolescents and youth with disabilities (AYPWDs) face multi-level barriers to sexual and reproductive health services, including poor socioeconomic status, lack of support, and negative community attitudes. Organisational barriers include healthcare worker maltreatment, communication difficulties, and breaches of confidentiality. |
| 2 | Kumi-Kyereme (2021) | Ghana | Quantitative | Mixed | Seven out of every 10 respondents had ever used SRHS. The proportion was higher among males compared with females. YPWD in the coastal and middle zones were less likely to have ever utilised SRHS compared with those in the northern ecological zone. The blind pupils or students were more likely to have ever utilised SRHS than the deaf. |
| 3 | Binu et al. (2018) | Ethiopia | Quantitative | Mixed | A study of 739 secondary school youth found a 21.2% sexual and reproductive health (SRH) service utilisation rate, with key drivers including discussions with health workers (AOR 3.0), previous STI symptoms (AOR 2.6), and sexual experience (AOR 5.9). Conversely, information from school teachers (AOR 0.36) was a negative predictor, while barriers included privacy concerns, timing, and parental prohibition. |
| 4 | Mesiäislehto et al. (2021) | Tanzania | Qualitative | Mixed | The results demonstrate that discrimination affects access at different phases of care-seeking, that the affectionate behaviour of providers is a central enabler of access, and that for this population, access relies on a collective effort. |
| 5 | Burke et al. (2017) | Senegal | Qualitative | Mixed | Young people with disabilities exhibit low awareness and usage of sexual and reproductive health (SRH) services despite a clear need, often facing restricted access due to reliance on others for transportation. Key barriers include financial constraints, negative provider attitudes, physical accessibility issues, and a lack of confidentiality, with reports of rape, particularly among women with hearing impairments. |
| 6 | Senayah et al. (2019) | Ghana | Quantitative | Deaf | Poor quality due to severe communication barriers and no sign language. |
| 7 | Kassa et al. (2016) | Ethiopia | Quantitative | Mixed | Radio/TV are main info sources; quality poor due to inaccessibility. |
| 8 | Schenk et al. (2020) | Multi-country | Qualitative | Mixed | HIV services rated poor; compounded stigma and misinformation. |
| 9 | Mesiäislehto et al. (2024) | Ghana | Qualitative | Mixed | The study findings indicated discrimination affected access at different phases of care-seeking, that affectionate behaviour of providers is a central enabler of access, and that for this population access relies on a collective effort |
| 10 | Abimanyi-Ochom (2017) | Uganda | Mixed-methods | Mixed | Significant knowledge gap; youth with disabilities have less HIV info. |
| 11 | Obasi et al. (2019) | Ghana | Quantitative | Mixed | Weak provider engagement and insufficient contraceptive info. |
| 12 | Agu et al. (2024) | Nigeria | Quantitative | Mixed | Harsh/unfriendly environments and discrimination deter utilisation. |
| 13 | Ngilangwa et al. (2016) | Tanzania | Mixed-Methods | Mixed | Overall, 317 (79.6%) had SRH rights knowledge. The leading sources of SRH rights information were peer educators (36.7%) and radio (22.8%). Awareness regarding laws and policies related to SRH right was 55.1%. However, young people living with HIV and school truants had more access to SRHR education and services than the other youth groups (χ2 30.69, p< 0.0001). The qualitative study revealed that parents and teachers perceived themselves to be incompetent in providing SRH information to their youngsters despite of being mostly trusted |
| 14 | Diribsa et al. (2022) | Ethiopia | Quantitative | Mixed | Identifies determinant factors of SRH service use among AYPWDs. |
| 15 | Pincock et al. (2023) | Ethiopia | Qualitative | Mixed | Evaluates Comprehensive Sexuality Education (CSE) for marginalised youth. |
| 16 | Bukuluki et al. (2023) | Uganda | Quantitative | Mixed | Examines sexual behaviour and utilisation specifically among AYPWDs. |
